# Supplementary material for: Wnt/PCP controls spreading of Wnt/β-catenin signals by cytonemes in vertebrates
Source: eLife. 2018 Jul 31;7:e36953. doi: 10.7554/eLife.36953 (PMC6086664; doi:10.7554/eLife.36953)
Supplement: Transparent reporting form [file elife-36953-transrepform.docx]

***eLife’s* transparent reporting form**

We encourage authors to provide detailed information *within their submission* to facilitate the interpretation and replication of experiments. Authors can upload supporting documentation to indicate the use of appropriate reporting guidelines for health-related research (see [EQUATOR Network](http://www.equator-network.org/%20)), life science research (see the [BioSharing Information Resource](https://biosharing.org/)), or the [ARRIVE guidelines](http://www.plosbiology.org/article/info:doi/10.1371/journal.pbio.1000412) for reporting work involving animal research. Where applicable, authors should refer to any relevant reporting standards documents in this form.

If you have any questions, please consult our Journal Policies and/or contact us: [editorial@elifesciences.org](mailto:editorial@elifesciences.org).

**Sample-size estimation**

- You should state whether an appropriate sample size was computed when the study was being designed
- You should state the statistical method of sample size computation and any required assumptions
- If no explicit power analysis was used, you should describe how you decided what sample (replicate) size (number) to use

Please outline where this information can be found within the submission (e.g., sections or figure legends), or explain why this information doesn’t apply to your submission:

An appropriate sample size was computed when the study was being designed.

Cumulative filopodia length or average number of filopodia per 10 cells per transfection reactions or in 5 zebrafish embryos with indicated constructs were measured.

Analysis of cell roundness ws carried out by measuring the width/length ratio of 20 notochordal cells in 5 zebrafish embryos.

RTqPCR or reporter assays show relative ΔCt value of target gene expression normalized to control group (e.g. actb1 expression).

All experiments were done in 3 independent triplicates (meaning three independent experiments, preferably executed in three consecutive weeks).

**Replicates**

- You should report how often each experiment was performed
- You should include a definition of biological versus technical replication
- The data obtained should be provided and sufficient information should be provided to indicate the number of independent biological and/or technical replicates
- If you encountered any outliers, you should describe how these were handled
- Criteria for exclusion/inclusion of data should be clearly stated
- High-throughput sequence data should be uploaded before submission, with a private link for reviewers provided (these are available from both GEO and ArrayExpress)

Please outline where this information can be found within the submission (e.g., sections or figure legends), or explain why this information doesn’t apply to your submission:

All experiments were done as independent triplicates. This means the entire experiment (e.g. collection of zebrafish embryos, experimental manipulation, measurement by high-resolution and validation) was performed in three consecutives and independent approaches.

**Statistical reporting**

- Statistical analysis methods should be described and justified
- Raw data should be presented in figures whenever informative to do so (typically when N per group is less than 10)
- For each experiment, you should identify the statistical tests used, exact values of N, definitions of center, methods of multiple test correction, and dispersion and precision measures (e.g., mean, median, SD, SEM, confidence intervals; and, for the major substantive results, a measure of effect size (e.g., Pearson's r, Cohen's d)
- Report exact p-values wherever possible alongside the summary statistics and 95% confidence intervals. These should be reported for all key questions and not only when the p-value is less than 0.05.

Please outline where this information can be found within the submission (e.g., sections or figure legends), or explain why this information doesn’t apply to your submission:

Statistical analysis was carried out by using unpaired two-sample t-tests. Two-sample t-tests for a difference in mean involved independent samples or unpaired samples. The independent samples t-test was used when two separate sets of independent and identically distributed samples are obtained, one from each of the two populations being compared. Significance level as indicated: ***=P<0.001, **=P<0.01, *=P<0.05

Presentation of data:

Data points are presented as a boxplot. Centre lines show the median; box limits indicate the 25th and 75th percentiles as determined by R software; whiskers extend 1.5 times the interquartile range from the 25th and 75th percentiles, outliers are represented by dots.

(For large datasets, or papers with a very large number of statistical tests, you may upload a single table file with tests, Ns, etc., with reference to sections in the manuscript.)

**Group allocation**

- Indicate how samples were allocated into experimental groups (in the case of clinical studies, please specify allocation to treatment method); if randomization was used, please also state if restricted randomization was applied
- Indicate if masking was used during group allocation, data collection and/or data analysis

Please outline where this information can be found within the submission (e.g., sections or figure legends), or explain why this information doesn’t apply to your submission:

Samples were allocated randomly within experimental groups (blind tests).

**Additional data files (“source data”)**

- We encourage you to upload relevant additional data files, such as numerical data that are represented as a graph in a figure, or as a summary table
- Where provided, these should be in the most useful format, and they can be uploaded as “Source data” files linked to a main figure or table
- Include model definition files including the full list of parameters used
- Include code used for data analysis (e.g., R, MatLab)
- Avoid stating that data files are “available upon request”

Please indicate the figures or tables for which source data files have been provided:

We will make the data publicly available, unless there are reasons to restrict access. We will deposit datasets into domain-specific public archives such as GenBank or Protein Data Bank.
